# Cell-free fat extract-loaded microneedles attenuate inflammation-induced apoptosis and mitochondrial damage in tendinopathy

**DOI:** 10.1016/j.mtbio.2023.100738

**Published:** 2023-08-01

**Authors:** Tianyou Kan, Zhaoyang Ran, Lin Sun, Xu Jiang, Lingli Hou, Yiqi Yang, Zhuoxuan Jia, Wenjie Zhang, Liao Wang, Mengning Yan, Kai Xie

**Affiliations:** a Shanghai Key Laboratory of Orthopedic Implants, Department of Orthopedic Surgery, Shanghai Ninth People's Hospital, Shanghai Jiao Tong University School of Medicine, Shanghai, 200125, China; b Shanghai Institute of Precision Medicine, Shanghai Ninth People's Hospital, Shanghai Jiao Tong University School of Medicine, Shanghai, 200125, China; c Shanghai Key Laboratory of Tissue Engineering, Department of Plastic and Reconstructive Surgery, Shanghai Ninth People's Hospital, Shanghai Jiao Tong University School of Medicine, Shanghai, 200011, China

**Keywords:** Tendinopathy, Cell-free fat extract, Microneedle, Anti-apoptosis, Tumor necrosis factor

## Abstract

Existing clinical treatments for tendinopathy mainly focus on reducing pain, whereas inhibiting or reversing disease progression remains challenging. Local therapeutic drugs, such as glucocorticoids, cause adverse effects on the metabolism of tendon tissues and injection-related complications. Therefore, new administration modalities for tendinopathy need to be developed. In this study, we designed a hydrogel-based microneedle (MN) system for the long-term transdermal delivery of our novel biological cell-free fat extract (CEFFE) to treat tendinopathies. We found that CEFFE-loaded MNs (CEFFE-MNs) had good biosafety and inhibited lipopolysaccharide (LPS)-induced apoptosis and matrix degradation in Achilles tendon cells of rats. The Achilles tendons of rats returned to their maximum mechanical strength after applying CEFFE-MNs. The administration of CEFFE-MNs had better anti-apoptosis and tendon repair-promoting effects than CEFEF injections *in vivo*. Transcriptome sequencing indicated that the anti-apoptosis effect of CEFFE-MNs was highly related to tumor necrosis factor (TNF) signaling. CEFFE-MNs inhibited the expression of TNF, TNF receptor 1, and downstream nuclear factor-kappa B signaling. Additionally, CEFFE-MNs rescued LPS-induced mitochondrial dynamics in tendon cells via the TNF-Drp1 axis. Our study reports a novel CEFFE-MN system that exhibits long-term anti-inflammatory and anti-apoptotic effects, suggesting it as a new treatment route for tendinopathy with broad clinical translation prospects.

## Graphical abstract

Cell-free fat extract (CEFFE) is an acellular fluid extracted from human subcutaneous adipose tissue without cellular components and lipid residues. Dissolving microneedles (MNs) are composed of natural or synthetic polymers that are biodegradable and biocompatible. We used GelMA and PVA to wrap CEFFE and load it into MNs to obtain patches. The CEFFE-MNs were applied in Achilles tendinopathy rats, and CEFFE-MNs had better anti-inflammatory and tendon repair-promoting effects *in vivo* than *in vitro*. The underlying molecular mechanism relies on the inhibition of the TNF/NF-κB signaling pathways.

**Figure:**
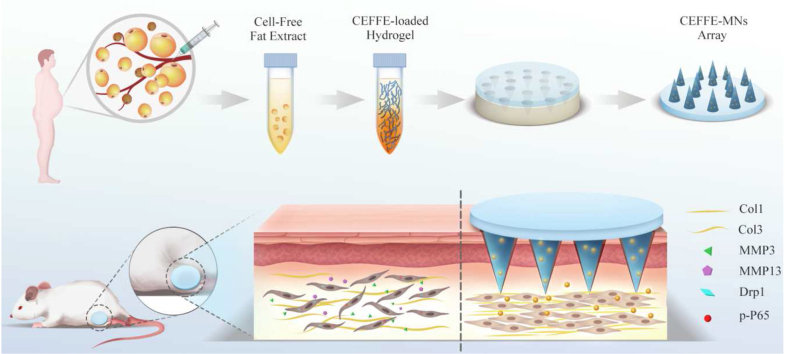

## 1 Introduction

Tendinopathy caused by injury and chronic inflammation leads to pain and tendon dysfunction. Since the early 2000s, the incidence of tendinopathy has been increasing worldwide, resulting in long-term or permanent functional impairment in athletic and non-athletic individuals of all ages [1,2]. The prevalence of tendinopathy in the population is approximately 5.9% whereas that of Achilles tendon disease in athletes is as high as 23.9% [3]. Pathological changes in the tendon are characterized by the abnormal microstructure, composition, and cellular structure of the tendon. Specifically, the inflammatory reaction of the diseased tendon, replacement of collagen I (Col 1) with collagen III (Col 3), and disordered arrangement of collagen lead to a decrease in the biomechanical strength of the tendon [1].

Current treatments for tendinopathy are local or systemic and mainly focus on reducing pain and improving function, with few treatments targeting the pathogenic mechanism. The three types of local treatments, namely the use of common drugs (glucocorticoids), biological agents (platelet-rich plasma [PRP]), and stem cell therapy, have several limitations, which limit their effectiveness. Moreover, treatment-related complications may occur because of multiple injections, and repeated corticosteroid injections may impair the physiological healing response of local tissues and promote disease progression [4,5]. Although PRP is recognized as a local treatment for tendinopathy, there is still a lack of high-level research evidence to prove its effectiveness in treating Achilles tendinopathy [1,6]. Additionally, cell therapy, including progenitor, stem, or autologous tendon cells, for treating Achilles tendinopathy has some limitations. Donor specificities, such as age and sex, significantly affect stem cell potential, including cell proliferation, differentiation, and the ability to promote angiogenesis and prevent apoptosis. Moreover, cell culture techniques have limitations such as the safety and quality of cell expansion *in vitro* and phenotypic, functional, and genetic instability [7,8].

Cell-free fat extract (CEFFE) is an acellular fluid extracted from human subcutaneous adipose tissue without cellular components and lipid residues and has shown no immunogenicity, good biosafety, and significant anti-aging effects and antioxidant activity in our previous studies [[9], [10], [11]]. CEFFE ameliorates osteoporosis by inhibiting reactive oxygen species (ROS) formation and reducing osteocyte apoptosis in osteoporotic mice [9]. CEFFE also promotes wound healing via the increased expression of vascular endothelial growth factor, Col 1, and Col 3 [10]. Therefore, CEFFE is promising for the treatment of various diseases, especially inflammatory diseases, owing to its lack of immunogenicity and antioxidant effects. However, the effect of CEFFE on tendinopathy remains unknown. Moreover, previous studies using a local injection of CEFFE revealed that the growth factors in CEFFE are rapidly degraded and unstable *in vivo* [11]. Therefore, a suitable mode of CEFFE transport that maintains its biological activity for a long time is required to improve its utilization and efficiency and avoid the side effects of repeated injections.

Dissolving microneedles (MNs) can penetrate the epidermis and enter the internal tissue in a painless, non-invasive, and non-infectious way [12]. MNs are composed of natural or synthetic polymers that are biodegradable, biocompatible, and dissolvable. Owing to these advantages, MNs have been used to deliver small molecular drugs, proteins, and cytokines across tissues [13]. CEFFE encapsulated in the polymer matrix is continuously released, which enhances its release and permeability. In the present study, we hypothesized that CEFFE may have potential therapeutic effects on chronic Achilles tendinopathy and aimed to develop a novel method using CEFFE-MNs for the local treatment of tendinopathy. We encapsulated CEFFE in a bioactive hydrogel to prepare an MN drug delivery system. CEFFE-loaded MNs (CEFFE-MNs) were sufficiently mechanically robust to penetrate the stratum corneum and reach the dermis. We detected the effect of CEFFE-MNs on tendon cell apoptosis *in vitro*, applied them in a rat model of Achilles tendinopathy *in vivo*, and tested the tensile modulus of the Achilles tendon. Moreover, we investigated the underlying molecular mechanism and mitochondrial function of CEFFE using transcriptome sequencing.

## 2 Materials and methods

### 2.1 Materials and reagents

Gelatin methacryloyl (GelMA), polyvinyl alcohol (PVA), and lithium phenyl (2,4,6-trimethylbenzoyl) phosphinate (LAP) components of tips and hyaluronic acid methacryloyl (HA) components of the base of MNs were purchased from Engineering for Life (Suzhou, China). GelMA was prepared from gelatin and methacrylic anhydride with an amino substitution of 90%. Low-glucose Dulbecco's modified Eagle medium (DMEM/low glucose) was obtained from Hyclone Laboratories Inc. (Logan, UT, USA), fetal bovine serum (FBS) from Sigma Aldrich (St. Louis, MI, USA), and l-glutamine and penicillin-streptomycin from Sangon Biotech (Shanghai, China). All primers for quantitative polymerase chain reaction (qPCR) were sourced from Sangon Biotech (Shanghai, China), and all proteins for western blotting were sourced from Abcam (Cambridge, UK). Dojindo Molecular Technology, Inc. (Kumamoto, Japan), provided the Cell Counting Kit-8 (CCK-8). Lipopolysaccharide (LPS) was purchased from Kingmorn Life Sciences (Shanghai, China). A phosphate-buffered (PBS) solution was prepared, and deionized water was used throughout the experiments.

### 2.2 Animals and cell culture

The Committee of Ethics in Animal Experiments at the Shanghai Jiao Tong University School of Medicine approved the animal experiments (SH9H-2022-A878-1). Male Sprague-Dawley rats (300 − 400 g, Shanghai SIPPR BK Laboratory Animals Ltd., Shanghai, China) were housed individually in a temperature-controlled animal facility with a 12-h light/dark cycle and free access to food and water.

Achilles tendons were harvested from rats and tendon cells were obtained by digestion of tendons with 3 mg/mL of collagenase I solution. In addition, tendon cells were cultured in DMEM/low glucose with 10% FBS, 1% l-glutamine, and 1% penicillin-streptomycin in a humidified atmosphere (37 °C, 5% CO_2_). The medium was changed every 3 days. Tendon cells within passage 5 were used in the experiments.

### 2.3 CEFFE preparation

The CEFFE provided by the SEME CELL Co. Ltd. (Shanghai, China) was extracted as described previously [11]. In brief, the fresh fat obtained from healthy volunteers was mechanically emulsified after centrifugation. The third aqueous layer was retained after re-centrifugation, filtered using a 0.22 μm filter, and stored at −80 °C. The CEFFE protein concentration was 5000 μg/mL, as detected using a bicinchoninic acid (BCA) protein assay kit (Beyotime Biotechnology, Shanghai, China).

### 2.4 Design and characterization of MNs

A glochis-like MN array was designed, and a negative frame was made of silica gel. Briefly, accurate rectangular pyramids and cylinders were fashioned carefully and welded by poly(methyl methacrylate). The corresponding negative frame was then cast and used to fabricate a circular MN array containing 385 MNs. The height of each MN was 700 μm, the base diameter was 270 μm, and the distance between two adjacent MNs was 700 μm.

The mechanical properties of microneedles are determined by a general purpose tester. When the upper plate initially touches the microneedle tip, the distance between the upper and lower plates is zero. The lower plate moves up to the MNs at a constant speed of 0.1 mm/min until the needle bends and breaks. The compression force is recorded. The yield stress of a single stitch is calculated as follows: σ = *F/S*, where σ represents the yield stress, *F* represents the compressive force (total force/number of needles), and *S* represents the bottom area of a single needles. Skin sections are used to verify the effect of microneedles.

### 2.5 Production of the CEFFE-MNs array

The MN array was produced using a negative frame. Specifically, each needle cavity was filled with tip and body solution, prepared by mixing 0.15 g of GelMA, 0.1 g of PVA, and different amounts of CEFFE (50, 100, 200, 500, and 1000 μg) dissolved in 0.25% LAP into 1 mL solution with 20 μL protease inhibitor. HA (4%) was dissolved in PBS as the base solution. Next, 500 μL of the tip and body solution was added to the negative frame using a pipette, filling the cavities via vacuum, dried, and concentrated at 30 °C. The redundant solution was then removed, and the tips were solidified under UV irradiation (405 nm) for 60 s. Next, 300 μL of the base solution was added to cover the tips, centrifuged to eliminate bubbles, and dried at 30 °C overnight. Finally, the resultant MNs were demolded by pulling the negative frame. These MNs were stored sealed and in dry conditions at 4 °C and used as soon as possible.

### 2.6 Preparation of CEFFE-MNs extracts

CEFFE-MNs extracts were prepared according to ISO 10993.12 to carry out the *in vitro* cell experiments. Specifically, MNs loaded with different concentrations of CEFFE were placed in DMEM/low glucose containing 10% FBS, 1% l-glutamine, and 1% penicillin-streptomycin in a humidified atmosphere (37 °C, 5% CO_2_) for 72 h to obtain the MN extracts containing CEFFE at different concentrations.

### 2.7 In vitro cell experiments

Tendon cells were seeded in 6-well plates at 3.0 × 10^5^ cells per well and divided into control, LPS, and CEFFE therapy groups. Each group of cells was cultured in DMEM/low glucose supplemented with 10% FBS, 1% l-glutamine, and 1% penicillin-streptomycin until adhesion. In addition, LPS (50 ng/mL) was used as a stimulation factor of the aseptic inflammatory process of tendon cells *in vitro*. Briefly, tendon cells of the LPS and CEFFE therapy groups were incubated with 50 ng/mL of LPS for 48 h; the cells of the LPS group were treated with normal culture medium, and the cells of the CEFFE therapy group were treated with CEFFE-loaded MN extracts for 48 h. After treatment, each group of cells was used for subsequent experiments.

### 2.8 Cell viability assay

Tendon cells (2000/well) were seeded in 96-well plates with corresponding culture medium and cultured at 37 °C with 5% CO_2_. After 48 h of incubation, LPS (50 ng/mL) and MNs loaded with 0, 50, 100, 200, 500, or 1000 μg of CEFFE were added to the culture medium. After 48 h of incubation, cell viability was determined using the CCK-8 assay according to the manufacturer's instructions (n = 5) to determine the optimal concentration of the extract for subsequent experiments. Cells cultured in DMEM/low glucose supplemented with 10% FBS, 1% l-glutamine, and 1% penicillin-streptomycin were used as the control group.

### 2.9 Degradation and release experiment

The degradation of CEFFE-MNs *in vitro* was observed by immersion in simulated body fluid (SBF). Briefly, all CEFFE-MNs were lyophilized. After determining the initial weight, CEFFE-MNs were placed on a cell filter with an aperture of 70 μm. Next, the cell filter was placed on a 6-well plate containing 5 mL of SBF so that CEFFE-MNs were completely immersed. Then, the CEFFE-MNs were removed at each set time point, washed with deionized water three times, freeze-dried, and weighed within 28 days.

The release experiment of CEFFE-MNs *in vitro* was observed by BCA protein assay kit. Briefly, all CEFFE-MNs were placed on a cell filter with an aperture of 70 μm. Next, the cell filter was placed on a 6-well plate containing 5 mL of SBF so that CEFFE-MNs were completely immersed. Then, the total protein concentration of the leaching solution was measured within 28 days.

### 2.10 RNA-sequencing and data analysis

Tendon cells of the LPS and CEFFE therapy groups were incubated with 50 ng/mL LPS for 48 h. The cells of the LPS group were treated with normal culture medium, and the cells of the CEFFE therapy group were treated with CEFFE for 48 h. Total RNA was isolated from different groups of primary tendon cells using TRIzol reagent (Thermo Fisher Scientific, Waltham, MA, USA), according to the manufacturer's instructions. Messenger RNA (mRNA) was then enriched using magnetic beads with oligo-dTs and used as the template for complementary DNA (cDNA) synthesis. After purification, the cDNA library was obtained by PCR enrichment. The fragment per kilobase million value of each gene was calculated for each cDNA sample using the FeatureCounts software and used as the gene expression value. Differential gene expression analysis was performed using the DESeq2 R package (version 1.16.1; https://www.r-project.org). Kyoto Encyclopedia of Genes and Genomes (KEGG) enrichment analysis was performed to determine the potential biological functions of the differentially expressed genes.

### 2.11 RNA extraction and quantitative real-time PCR (qRT-PCR)

Tendon cells were seeded in 6-well plates at 3.0 × 10^5^ cells per well. After treatment, total RNA was extracted from the cells in each group using TRIzol reagent (Thermo Fisher Scientific), according to the manufacturer's instructions. Reverse transcription was performed using the Supermix Kit (Bimake, Houston, TX, USA) and the resulting diluted cDNA was analyzed by qPCR using SYBR Green (Bimake). The qRT-PCR primers used in this study were designed using PrimerBlast (https://www.ncbi.nlm.nih.gov/tools/primer-blast/) and are listed in Table 1.

**Table 1:** Primers used in real-time PCR.

Target genes	Forward(5′-3)	Reverse(5′-3)
IL-1β	TGTCTGACCCATGTGAG	GCCACAGGGATTTTGTC
TNF-a	CTGGCGTGTTCATCCGTTCTCTAC	ACTACTTCAGCGTCTCGTGTGTTTC
TNFR1	GAACACCGTGTGTAACTGCC	ATTCCTTCACCCTCCACCTC
Nfkb1	TTCCTGATCCCGACAAGAACTG	CCCCCAGAGACCTCATAGTTGT
Gapdh	GATGCTGGTGCTGAGTATG	GTGGTGCAGGATGCATTGCT

### 2.12 Western blotting

Tendon cells were seeded in 6-well plates at 3.0 × 10^5^ cells per well. Briefly, tendon cells of the LPS and CEFFE therapy groups were incubated with 50 ng/mL LPS for 48 h and then treated with culture medium or CEFFE for 48 h, respectively. After treatment, total protein was collected from each group of tendon cells, and the cells were lysed in ice-cold RIPA buffer containing a protease inhibitor cocktail (Sigma-Aldrich) for 30 min. The lysates were centrifuged at 12,000 rpm for 20 min at 4 °C, and the supernatant was collected. The total protein concentration was quantified using a bicinchoninic acid protein assay kit (Beyotime Biotechnology) according to the manufacturer's protocol. Next, total protein was subjected to 15% sodium dodecyl sulphate-polyacrylamide gel electrophoresis, transferred to polyvinylidene difluoride membranes (Millipore, Billerica, MA, USA), blocked with 5% bovine serum albumin (BSA) for 1 h at 25 °C, and then incubated with interleukin (IL)-1β, cyclooxygenase 2 (COX2), Col1a, Col3, matrix metalloproteinase (MMP)3, MMP13, B-cell lymphoma 2 (Bcl-2) and its associated proteins Bad and Bax, Cleaved-caspase 3, dynamin-related protein 1 (Drp1), mito-fusion 1 (Mfn 1), TNF-α, TNF Receptor 1 (TNFR1), IκBα, p-IκBα, P65, p-P65, and β-actin at 4 °C overnight. After that, the mixture was conjugated with a secondary peroxidase antibody for 1 h. Subsequently, the membranes were incubated with anti-rabbit or anti-mouse immunoglobulin G (CST, Danvers, MA, USA) for 1 h at room temperature. Protein bands were visualized using an Odyssey infrared imaging system (LI-COR Biosciences, Lincoln, NE, USA).

### 2.13 Flow cytometry

Apoptosis of tendon cells induced by LPS was determined by flow cytometry using an annexin V/propidium iodide (PI) apoptosis detection kit (Yeasen Biotechnology Co., Shanghai, China). Briefly, tendon cells were stimulated for apoptosis with LPS, and the experimental group was incubated with CEFFE for 48 h. Next, the cells and culture medium were harvested, washed twice with cold PBS, and labeled with fluorescein isothiocyanate annexin V and PI in binding buffer. The cells were then subjected to flow cytometry using the BD LSR Fortessa system (BD, Franklin Lakes, NJ, USA) to detect the fluorescence intensity of the cells. Finally, the experiment was repeated thrice, and the apoptosis rate (%) of each group was calculated.

### 2.14 ROS assay

The levels of ROS in the different groups of tendon cells were detected using the fluorescence probe dichlorodihydrofluorescein-diacetate (DCFH-DA) (Beyotime Biotechnology). First, DCFH-DA was diluted in a serum-free culture medium at a ratio of 1:1000 to a final concentration of 10 μM. Then, the cell culture solution was removed from the well plate, 1 mL of diluted DCFH-DA was added to each well, and the plate was incubated for 20 min at 37 °C. Rosup (a ROS inducer) was added to the positive control. Finally, the cells were washed three times with serum-free cell culture medium to remove DCFH-DA, which did not enter the cells, and observed under a fluorescence microscope (Leica Microsystems, Wetzlar, Germany).

### 2.15 Mitochondrial membrane potential assay

The JC-1 fluorescent probe (Beyotime Biotechnology) was used to detect the mitochondrial membrane potential of tendon cells in each group. First, the JC-1 staining solution was prepared according to the manufacturer's instructions, and the cells were treated with 10 μm carbonyl cyanide-m-chlorophenylhydrazone (CCCP, a mitochondrial uncoupler) for 20 min as a positive control. Next, the JC-1 probe was loaded using the following method: the culture medium was removed from the Petri dish and washed with PBS, 1 mL cell culture medium was added, and the JC-1 staining working solution (1 mL) was added. After incubation at 37 °C for 20 min, the supernatant was removed, and the cells were washed twice with JC-1 staining solution. Finally, the cell culture medium (2 mL) was added to the Petri dish and observed under the fluorescence microscope.

### 2.16 Mitochondrial tracker assay

The mitochondria in the tendon cells of each group were specifically stained with MitoTracker Red CMXRos and Mito-Tracker Green (Beyotime Biotechnology). First, the Mito-Tracker Red CMXRos and Mito-Tracker Green working solutions were prepared according to the manufacturer's instructions. Then, the cell culture medium was removed from each Petri dish, the prepared working solutions were added, and cells were incubated at 37 °C for 30 min. After this period, the working solutions were removed from the Petri dish and fresh cell culture medium was added. After incubation at 37 °C, the mitochondria in the cells were observed under a fluorescence microscope.

### 2.17 Establishment of achilles tendinopathy rats

Twenty-four 12-week rats, with an average weight of 400 g, were used to establish Achilles tendinopathy models. After environmental adaptation for 1 week, during which the rats had ad libitum access to food and water, the animals were injected with 60 μL of collagenase I solution (5 mg/mL) in the right hind leg one time, except for the control group.

### 2.18 In vivo animal experiment

The rats were divided into four groups: control, tendinopathy, CEFFE-injected, and CEFFE-MNs (n = 6 per group). The rats in the control and tendinopathy groups received no treatment. In contrast, each rat in the CEFFE-injected group received 100 μg CEFFE by injection, while that in the CEFFE-MNs group received CEFFE-loaded MN arrays. After 28 days, the Achilles tendons of rats in all groups were extracted, and three tendons from each group were prepared for eosin and hematoxylin (HE), Masson, Sirius, and immunohistochemical staining assays. Briefly, tendon tissues were dehydrated in a graded ethanol series and embedded in paraffin before being sliced into 5-μm pieces. Next, these sections were dewaxed with xylene and transferred from ethanol to deionized water. After the antigen retrieval process, the sections were subjected to different staining and immunohistochemistry procedures for detecting Col1, Col3, MMP3, MMP13 and Cleaved-caspase3.

### 2.19 Biomechanical testing

Three Achilles tendons were selected for biomechanical tests in each group. After removing the surrounding muscles and blood vessels, the Achilles tendon were fixed. A 5 mm/min stretch was applied until the tendon was broken. The changes in tension and the maximum tension during the stretching process were recorded.

### 2.20 Scanning electron microscopy (SEM) analysis

The morphology of MNs was observed using SEM. The prepared hydrogel MNs were sequentially frozen at −20 °C and −80 °C for 6 h and then lyophilized for 12 h. The substrate was then pasted on the sample stage, and each sample was coated with gold-palladium in a sputter coater (Quorum SC7620; Quorum, Laughton, UK) for 45 s. SEM observations were then carried out on a Gemini300 instrument (ZEISS, Oberkochen, Germany).

### 2.21 Transmission electron microscopy (TEM) analysis

Achilles tendon cells cultured *in vitro* and extracted from rats were observed by TEM. The centrifuged mass of adherent cells and the extracted tissue mass of the Achilles tendon (1 mm^3^) were fixed and dehydrated according to the sample preparation steps of TEM. Specifically, the samples were fixed with 2.5% glutaraldehyde for 2.5 h, washed in PBS (0.1 M, pH 7.0) three times (3 min each time), and then fixed with 1% osmic acid for 2 h. After washing another three times with 0.1 M PBS, cells were serially dehydrated in 30%, 50%, 70%, 85%, 95%, and 100% ethanol (15 min in each step), and then dehydrated twice in 1:1 mixed ethanol plus acetone:pure acetone for 20 min each time. Cells were permeated with acetone:resin at 3:1, 1:1, 1:1, 1:1, and 1:1 for 1 h each time, infiltrated overnight with resin, and then embedded in fresh resin for 3 h. After resin blocks were polymerized at 37 °C for 8 h and 65 °C for 48 h, they were ultra-sliced (70–100 nm) and each ultra-slice was placed on a copper net with a carbon film. The ultra-slices were stained with uranyl acetate at 4 °C for 7 min and lead citrate for 3 min at 25 °C and then observed and photographed under TEM (Talos L120C, FEI; Thermo Fisher Scientific).

### 2.22 Statistical analyses

The results are expressed as the mean ± standard deviation. All data were analyzed using GraphPad Prism 9 (GraphPad Software Inc., San Diego, CA, USA), and differences were analyzed by one-way analysis of variance (ANOVA), followed by Tukey's post hoc test (group >2). All tests were performed with significance levels of *p < 0.05, **p < 0.01 and ***P < 0.001.

## 3 Results

### 3.1 Preparation of CEFFE-MNs

**Fig. 1:**
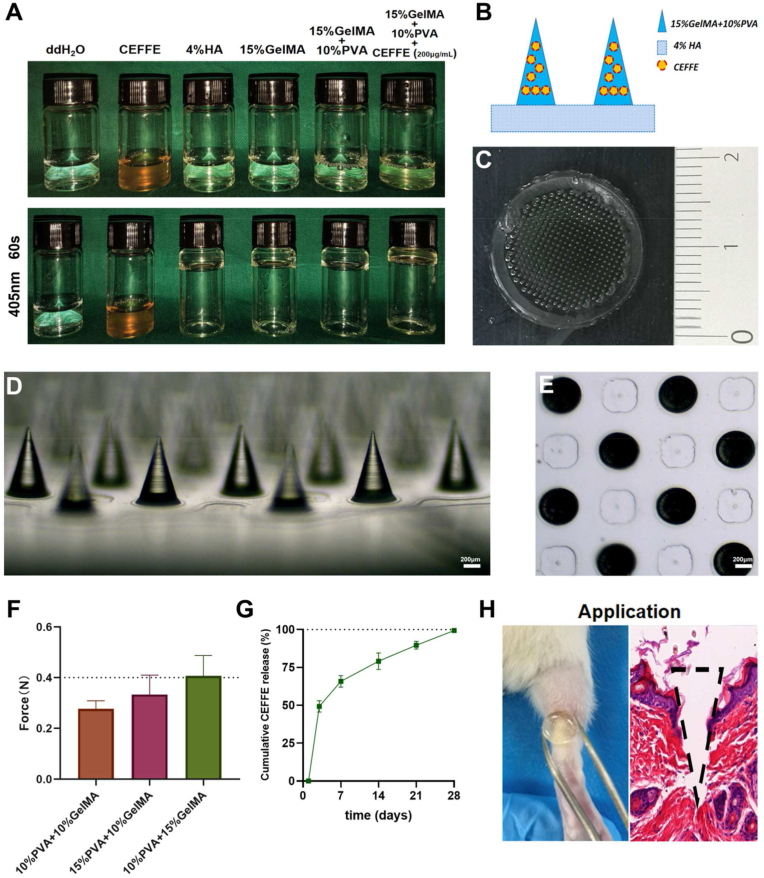
**Fabrication of CEFFE-MNs and Degradation *in vitro*.** (A) Gelling capacity of CEFFE-loaed MNs drug delivery system. (B) Schematic diagram of CEFFE-MNs structure. (C) General view of CEFFE-MNs. (D) The side-view of CEFFE-MNs. (E) The top-view of CEFFE-MN. (F) Mechanical strength testing of CEFFE-MNs. (G) CEFFE release rate at 1–28 days. (H) Application of CEFFE-MNs to animal experiments and the transdermal effect of CEFFE-MNs.

The manufacturing techniques using a negative die are described in the Methods section. GelMA, PVA, and HA with good biosafety and bioactivity were selected as biocompatible materials for the preparation of MNs owing to the basic requirements of these drug-carrying materials. To minimize the impact of the material on the effects of CEFFE and MNs, its extracts were used for the determination of the therapeutic concentration and subsequent *in vivo* and *in vitro* experiments. The CCK-8 assay indirectly indicated that hydrogel-coated 200 μg/mL CEFFE had the best anti-inflammatory effect (Figure S1A) and had good gelatinization performance (Fig. 1A). CEFFE-MNs were prepared using a negative mold. Finally, 4% HA was used as the substrate for the MNs and its swelling property favored efficient detachment of the substrate from the body of the MNs (Fig. 1B). The shaped MNs have sharp tips (Fig. 1C–E and S1B). For non-invasive treatments, MNs must have sufficient strength to penetrate the skin and then slowly degrade and release the drug during treatment [14]. Therefore, it is necessary to test the penetrability, degradation, and drug-release properties of MNs. The high amino substitution ratio of GelMA is conducive to its high mechanical strength after cross-linking (Fig. 1F and G). The mechanical strength of the MNs prepared with 15% GelMA and 10% PVA allowed them to penetrate the rat skin (Fig. 1H), after which the MNs were gradually degraded within 28 days (Figure S1C-E).

### 3.2 CEFFE-MNs promote collagen synthesis and inhibit inflammation-induced apoptosis

**Fig. 2:**
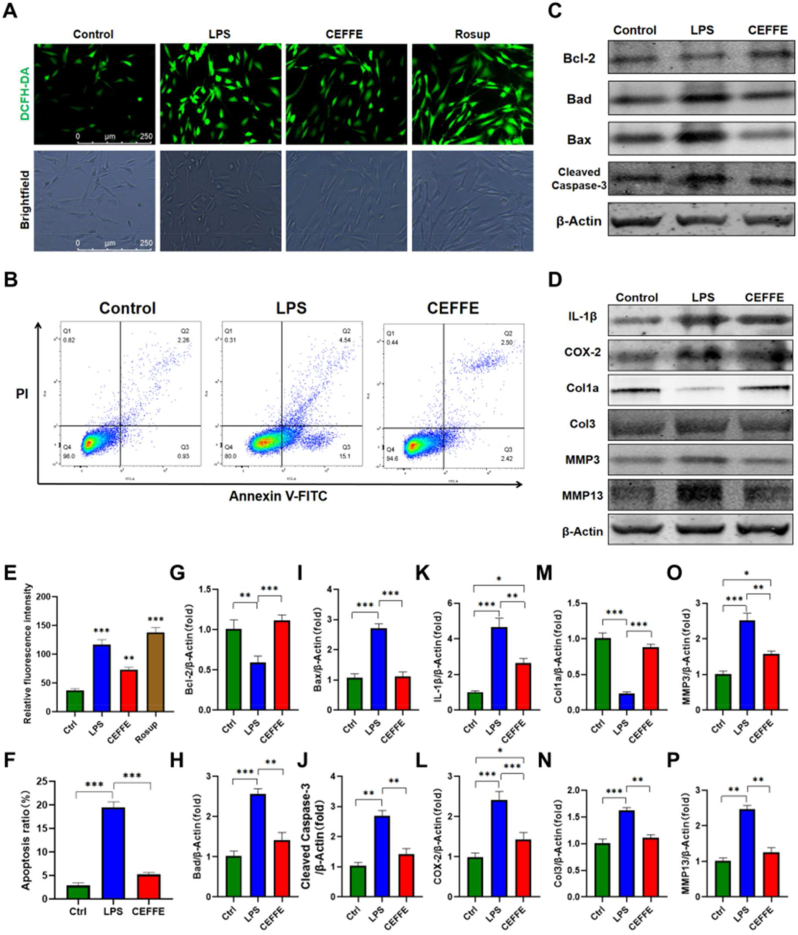
**CEFFE rescued LPS-induced oxidative stress, apoptosis and inflammation of tendon cells.** (A) Fluorescent green DCFH-DA staining and bright field imaging (magnification × 100). Scale bars, 250 μm. (B) Flow cytometry showed that CEFFE significantly decreased apoptosis of tendon cells induced by LPS. (C, D) LPS (50 ng/mL) were added to tendon cells to induce an inflammatory state, and CEFFE (200 μg/mL) treating the protein levels of Bcl-2, Bad, Bax, Cleaved-caspase 3, IL-1β, COX-2, Col1a, Col3, MMP3, MMP13 and β-Actin. (E) Fluorescent green DCFH-DA staining intensity quantification, calculated by ImageJ (N = 3). (F) Flow cytometry analysis of apoptosis statistics (N = 3). (G–P) The expression levels of proteins (Bcl-2, Bad, Bax, Cleaved Caspase-3, IL-1β, COX-2, Col1a, Col3, MMP3, MMP13) in the control group, LPS group and CEFFE group. (Data are shown as means ± SD, *P < 0.05, **P < 0.01, ***P < 0.001. (For interpretation of the references to color in this figure legend, the reader is referred to the Web version of this article.)

LPS-induced tendon cells were cultured for 48 h with extracts of CEFFE-MNs (200 μg/mL CEFFE). We observed significant oxidative stress and apoptosis in LPS-induced rat tendon cells. In addition, ROS overproduction in tendon cells was partially inhibited after treating LPS-induced tendon cells of rats with CEFFE-MNs (Fig. 2A, E). Moreover, by labeling tendon cells of rats with annexin-V and PI in each group and performing flow cytometry analysis, we found that, compared to the LPS group, the number of early and late apoptotic tendon cells in the CEFFE-MNs group was significantly reduced and was similar to that in the control group (Fig. 2B, F). Similar results were obtained in the western blot analysis of the expression of marker proteins of apoptosis; the expression of the anti-apoptotic protein Bcl-2 in the CEFFE-MNs group was increased compared to that in the LPS group, whereas the expression of the pro-apoptotic proteins Bad, Bax, and cleaved-caspase 3 decreased to a level similar to that in the LPS group (Fig. 2C, G-J). These results suggest that CEFFE-MNs rescued LPS-induced oxidative stress and apoptosis in tendon cells.

Western blot analysis also showed that LPS can stimulate the chronic inflammation of tendon cells *in vitro*. Specifically, the expression of IL-1 β and COX-2 was significantly upregulated in the LPS group after 50 ng/mL LPS stimulation for 48 h, and MMP3 and MMP13 expression levels were also increased. Compared to the LPS group, the expression of the inflammatory factors IL-1 β and COX-2 was decreased in the CEFFE-MN group. Col 1 expression was significantly upregulated and similar to that in the control group, whereas the expression levels of Col 3, MMP3, and MMP 13 were decreased (Fig. 2 D, K–P). These results indicate that CEFFE-MNs alleviate tendon inflammation *in vitro* and promote the synthesis of cellular matrix.

### 3.3 Effect of CEFFE-MNs on anti-inflammatory and restoration of maximum tensile strength of achilles tendon

**Fig. 3:**
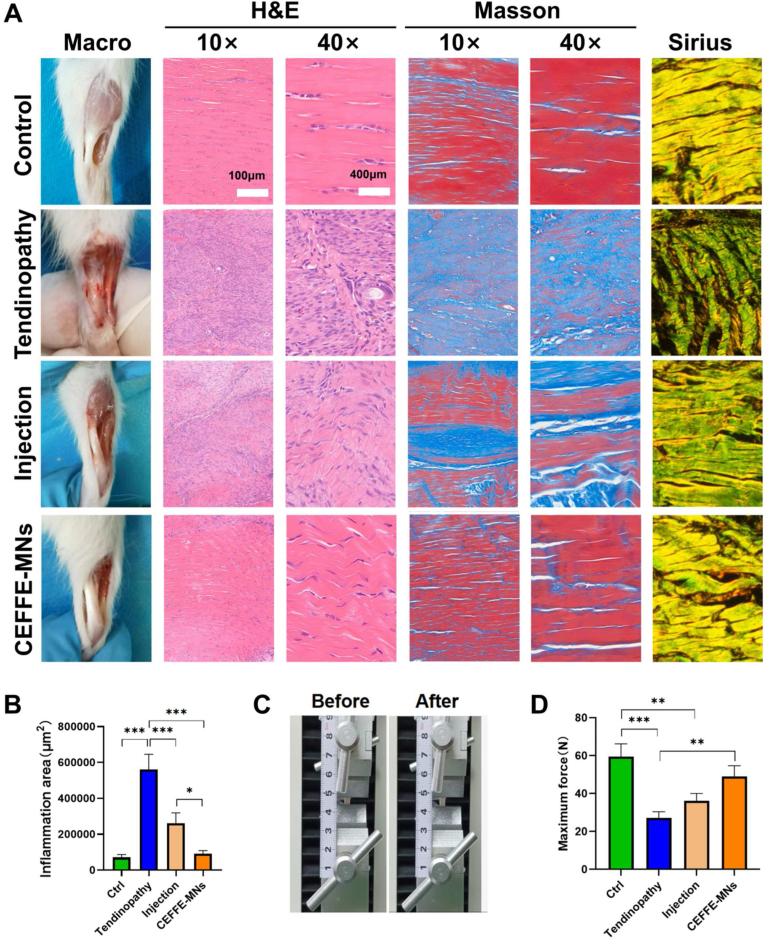
**Evaluation of the tendon inflammation and biomechanics of the CEFFE-MNs on AT rats.** (A) Macro, H&E staining of tendon, Masson staining and Sirius staining in the four groups (control, tendinopathy, Injection and CEFFE-MNs). (B) Total areas of inflammation in the four groups, calculated by ImageJ. (C) Tendon stretching test procedure. (B) Maximum force of tendon in the four groups (N = 3). Data are represented as the mean ± SD. *P < 0.05, **P < 0.01, ***P < 0.001.

We evaluated the effects of different CEFFE administration modes on the collagenase I-induced tendon inflammation of the control, tendinopathy, injection, and CEFFE-MN rat groups. Macroscopically, the Achilles tendon was rougher and more swollen in the tendinopathy group than in the control group. The isolation of the Achilles tendon facilitates the detection of its adhesion to the surrounding tissue after collagenase I induction. After 28 days of treatment with CEFFE-MNs t, the Achilles tendon was smooth, and its volume returned to normal (Fig. 3A). HE staining showed that the fibers in the tendinopathy group were disorderly arranged and infiltrated by inflammatory cells; after the simple injection of CEFFE, the arrangement of collagen fibers was tidier and more compact than that in the tendinopathy group, and this phenomenon was more pronounced in the CEFFE-MNs group. Collagenase I-induced tendon inflammation induced the thickening of the tendon as evidenced by collagen fiber swelling (increased blue-stained fibers after Masson staining) and extensive reduction of extracellular matrix (red-stained), which were reversed by treatment with CEFFE-MNs (Fig. 3B). Sirius staining revealed that most Col 1 in the control group (yellow-stained) was arranged neatly, whereas a large number of Col 3 fibers (green-stained) appeared in the tendinopathy group, and Col 1 in the injection and CEFFE -MN groups gradually increased Fig. 3A).

In addition, upon injury, the strength of the Achilles tendon decreases [15]. Therefore, we used mechanical testing of the Achilles tendon to explore the effect of MNs on its strength (Fig. 3C). The tensile test results of the Achilles tendon showed that the tendinopathy group had the lowest maximum tensile force whereas the CEFFE-MN group had a clearly increased maximum tensile force, which was no statistically significant difference from that in the control group (Fig. 3D). These results indicate that treatment with CEFFE-MNs can effectively improve the significantly reduced toughness, elasticity, and strength of the Achilles tendon after an inflammatory injury.

### 3.4 CEFFE-MNs improves collagen synthesis in achilles tendinopathy

**Fig. 4:**
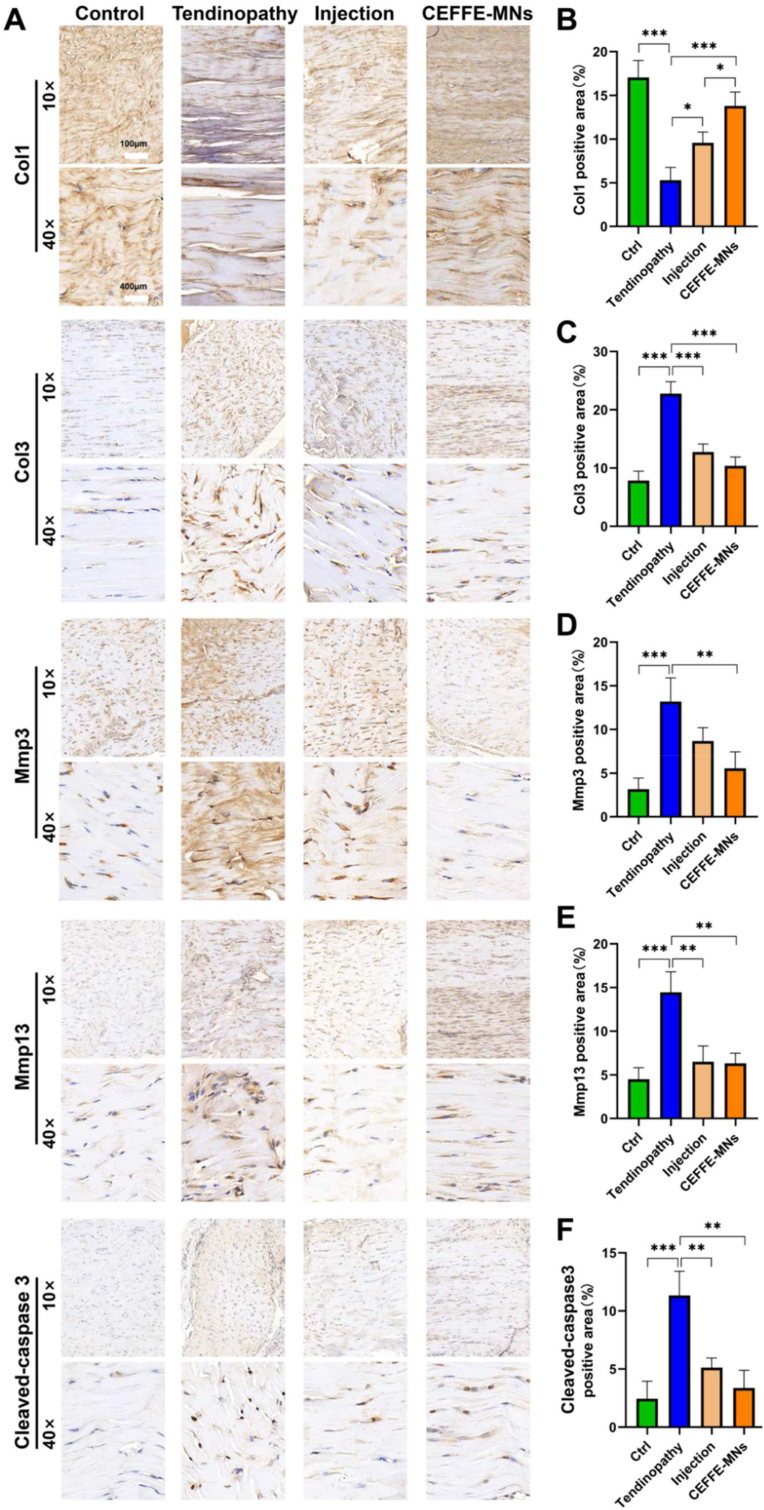
**Evaluation of the repair effect of the CEFFE-MNs on AT rats.** (A) Immunohistochemistry of Col1, Col3, Mmp3, Mmp13 and Cleaved-caspase 3 in the four groups. (B–F) The proportions of Col1, Col3, Mmp3, Mmp13 and Cleaved-caspase 3 positive areas in the four groups, calculated by ImageJ. Data are represented as the mean ± SD. *P < 0.05, **P < 0.01, ***P < 0.001.

Immunohistochemical analysis was performed on Achilles tendon tissue sections to demonstrate the repairing effect of CEFFE-MNs. TThe staining results of the apoptosis-related protein cleaved-caspase 3 also showed a lower staining intensity in the CEFFE-MN group than in the tendinopathy and injection groups. As Col 3 is less expressed in healthy tendons whereas Col 1 is highly expressed, in the control group, Col 1 expression levels were higher, the collagen fibers were arranged more neatly, and Col 3 expression levels were lower compared to the tendinopathy group (Fig. 4A–C). In addition, we observed significantly reduced and disordered collagen fibers and a large amount of inflammatory cell infiltration. Compared to the tendinopathy group, CEFFE-treated groups had significantly decreased Col 3 expression and significantly increased Col 1 expression, and this effect was more pronounced in the CEFFE-MNs group than in the injection group. The results of the Mmp3 and Mmp13 were similar, and the CEFFE-MNs group showed less positive staining than the tendinopathy and injection groups (Fig. 4A, D and E). The expression of Cleaced-caspase 3, a marker of apoptosis, was decreased (Fig. 4A, F). Concurrently, the results of HE staining of the organs of the rats in each group showed that neither the simple CEFFE injection nor the administration of CEFFE-MNs had obvious tissue toxicity to rats (Figure S2), indicating that the CEFFE-MN delivery system had better effect and biosafety. These results suggest that CEFFE injection can partially repair damaged Achilles tendon whereas the administration of CEFFE-MNs can further promote the therapeutic effect of CEFFE on collagenase I-induced tendon inflammation *in vivo*.

### 3.5 CEFFE-MNs rescues mitochondrial damage in achilles tendinopathy

**Fig. 5:**
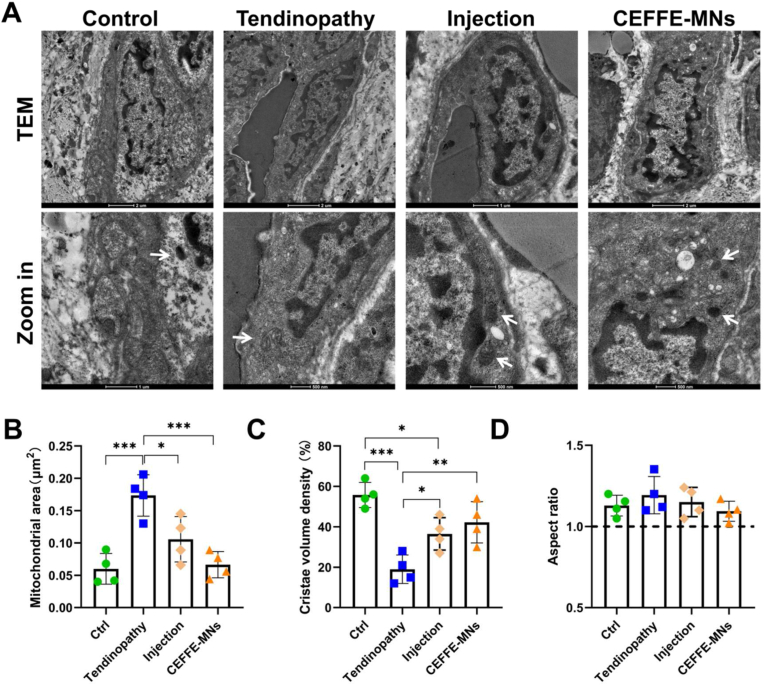
**Evaluation of the mitochondrial morphology of the CEFFE-MNs on AT rats.** (A) The TEM imaging of tendon and mitochondria in the four groups. (B) Mitochondrial area of tendon in the four groups, calculated by ImageJ. (C) Cristae volume density of tendon in the four groups, calculated by ImageJ. (D) Aspect ratio of tendon in the four groups, calculated by ImageJ. Data are represented as the mean ± SD. *P < 0.05, **P < 0.01, ***P < 0.001.

In addition, TEM demonstrated the therapeutic effect of CEFFE-MNs on the mitochondria of tendon cells. The morphology of the mitochondria in tendon cells in the CEFFE-Ms group was similar to that in the control group (Fig. 4E). (Fig. 5A). Specifically compared to the control group, type I collagenase induced mitochondrial swelling and cristae volume density reduction in rat Achilles tendon, and CEFFE-MNs could improve mitochondrial morphology (Fig. 5B and C). However, there was no significant difference in mitochondrial aspect ratio (Fig. 5D).

### 3.6 RNA-seq shows the inhibition of NF-κB by CEFFE-MNs

**Fig. 6:**
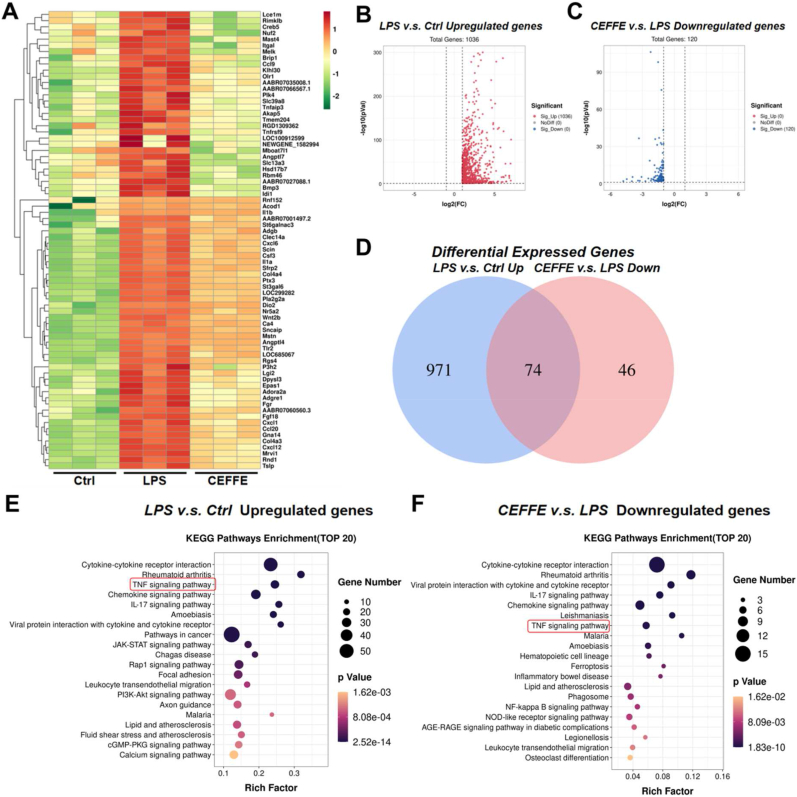
**CEFFE treatment inhibited TNF signaling pathway of LPS-induced tendon cells**. (A) Heatmap showing differentially expressed genes in the three groups (control, LPS and CEFFE group). Red: high expression levels. Green: low expression levels. (B,C) Volcano diagram showing the LPS and Ctrl groups upregulated genes and the CEFFE and LPS groups downregulated genes. two groups of differentially expressed genes, respectively. Red: the LPS and Ctrl groups upregulated genes. Blue: the CEFFE and LPS groups downregulated genes. (D) Veen diagram showing shared differentially expressed genes between the LPS and Ctrl groups upregulated genes and the CEFFE and LPS groups downregulated genes. (E,F) KEGG pathway enrichment analysis of the differentially expressed genes (between the LPS and Ctrl groups upregulated genes and betwwen the CEFFE and LPS groups downregulated genes. (For interpretation of the references to color in this figure legend, the reader is referred to the Web version of this article.)

The results of transcriptome sequencing for each group, visualized as heatmaps (Fig. 6A), volcano plots (Fig. 6B and C), and venn diagrams (Fig. 6D), show that compared to the control group, the tested genes were differentially upregulated after LPS addition and differentially downregulated after treatment with CEFFE-MN in LPS-treated tendon cells. KEGG enrichment analysis showed that LPS induced the upregulation of inflammation-related pathways, namely the TNF signaling pathway associated with inflammation and apoptosis (Fig. 6E and F) and the TNF/NF-κB pathway, which regulates the inflammatory response and apoptosis. The NF-κB signaling pathway plays a central role in inflammation, stress response, and cell survival. It is upregulated in tendinopathy and regulates cytokine production and apoptosis [1].

### 3.7 CEFFE-MNs alleviate inflammation in tendinopathy by inhibiting the TNF/NF-κB pathway

**Fig. 7:**
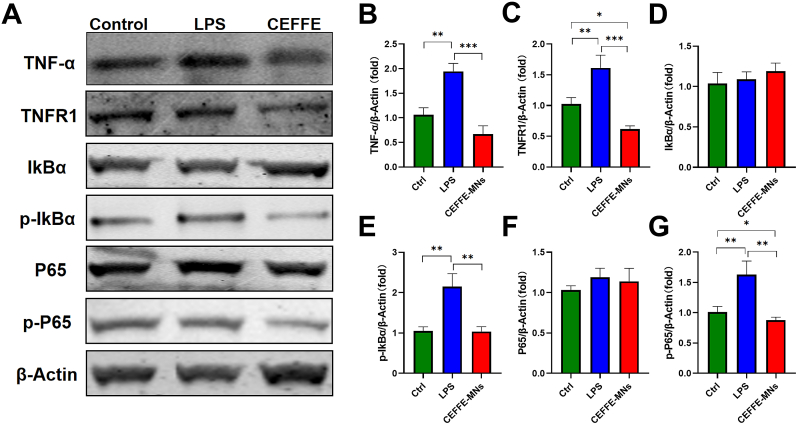
**CEFFE treatment inhibited TNF signaling pathway of LPS-induced tendon cells.** (A) TNF/NF-kB pathway related proteins levels of TNF-α, TNFR1, IkBα, p-IkBα, P65, p-P65 and β-Actin in the control group, LPS group and CEFFE group. (B–G) The expression levels of TNF/NF-kB pathway related proteins (TNF-α, TNFR1, IkBα, p-IkBα, P65 and p-P65) in the three groups. Data are shown as means ± SD, *P < 0.05, **P < 0.01, ***P < 0.001.

Therefore, we detected the expression of key proteins in the TNF/NF-κB pathways in tendon cells in each group using qRT-PCR *in vitro*, and the results were consistent with transcriptome sequencing results (Figure S3A-D). Moreover, based on the expression of key proteins in the NF-κB pathway, we found that LPS-induced tendon cell inflammation activated the NF-κB pathway, and this activity was inhibited after the application of CEFFE-MNs (Fig. 7A–G). Specifically, the expression of p-IκBα and p-P65 in tendon cells of the CEFFE-MN group was significantly lower than that in tendon cells of the LPS group and even lower than that in tendon cells of the control group (Fig. 7A,E and G).

### 3.8 CEFFE-MNs rescue mitochondrial damage in tendinopathy via TNF/NF-κB signaling

**Fig. 8:**
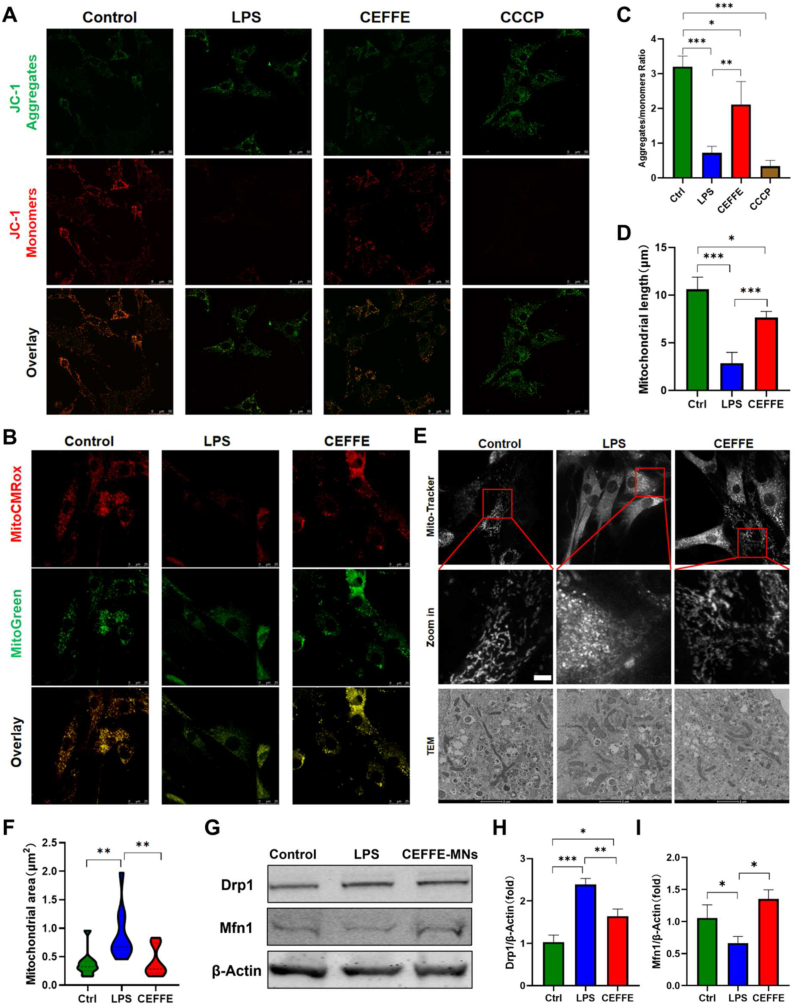
**The therapeutic effect of CEFFE on LPS-induced mitochondrial damage in tendon cells.** (A) Fluorescence image of JC-1 in the control group, LPS group and CEFFE group (magnification × 400). Scale bars 50 μm. (B) Quantification of fluorescence intensity of aggregates and monomer in each group (N = 3). (C) Fluorescence image of Mito-Tracker (MitoTracker Red CMXRos and MitoTracker Green) in each group. (D) Fluorescence image of Mito-Tracker and TEM to observe mitochondrial morphology in each group. (E) Mitochondrial length from fluorescence image of Mito-Tracker, calculated by ImageJ (N = 3). (F) Mitochondrial area from TEM, calculated by ImageJ (N = 3). (G) Mitochondrial fission protein levels of Drp1, Mfn1 and β-Actin. (H) The expression levels of Drp1 in each group. (I) The expression levels of Mfn1 in each group. Data are shown as means ± SD, *P < 0.05, **P < 0.01, ***P < 0.001. (For interpretation of the references to color in this figure legend, the reader is referred to the Web version of this article.)

CEFFE inhibited LPS-induced strong oxidative stress in tendon cells *in vitro*. Mitochondria are the center of cellular energy metabolism, playing an important role in maintaining cellular function in the process of apoptosis and regulating cellular redox balance. LPS-treated tendon cells generate excessive ROS, presumably causing damage to the mitochondria of tendon cells. Therefore, we evaluated the mitochondrial status of tendon cells based on their function and morphology. Fluorescence imaging of the mitochondrial membrane potential showed that LPS changed the mitochondria of tendon cells from multimers to monomers. After treatment with CCCP, the green fluorescence that indicated a decrease in membrane potential was significantly weakened, and the red multimer fluorescence was enhanced, resulting in a total fluorescence level similar to that of the normal mitochondrial membrane potential in the control group (Fig. 8A and B).

The mitochondrial membrane potential reflects mitochondrial function. Additionally, mitochondrial function is related to morphology. Therefore, we used mitochondrial probes to label the mitochondria of the tendon cells in each group. Mitochondrial trackers also reflect mitochondrial activity to a certain extent. Similarly, the signals of the two fluorescently labeled mitochondrial probes were significantly attenuated in the LPS group, indicating that LPS affects the mitochondrial activity of tendon cells. After treatment of LPS-induced tenocyte inflammation with CEFFE, the fluorescence intensity of the two mitochondrial probes recovered to levels close to those of the control group (Fig. 8C). Further observations of the labeled mitochondria revealed they were filamentous and network-like in the control group, reflecting normal mitochondrial function. However, LPS stimulation of the tendon cells significantly changed mitochondrial morphology, which showed rings and fragmentation; these LPS-induced mitochondrial damages decreased the membrane potential by increasing the permeability of the inner mitochondrial membrane and destroyed the normal mitochondrial network structure, affecting mitochondrial function. The mitochondrial morphological network structure was restored after treatment of the LPS-stimulated tendon cells with CEFFE (Fig. 8D). The comparison of mitochondrial length in tendon cells of each group also showed that the average mitochondrial length of cells in the LPS group was significantly lower than that in the control and CEFFE groups. The average mitochondrial length and area in the CEFFE group were also significantly higher than those in the LPS group but similar to the control group (Fig. 8E and F). Mitochondrial morphology is controlled by mitochondrial dynamics, including fusion and fission, which are responsible for different dyneins. Western blot analysis revealed that the expression of the mitochondrial fission protein Drp 1 in the LPS group was increased whereas it was relatively decreased in the CEFFE group (Fig. 8G and H)·In contrast, Mfn1 expression was up-regulated in the CEFFE group (Fig. 8G,I), indicating that CEFFE-MNs rescued LPS-induced mitochondrial damage in tendon cells.

## 4 Discussion

In the present study, we detected the effect of CEFFE on the apoptosis of tendon cells and found that CEFFE can inhibit inflammation-induced apoptosis. CEFFE- MNs were applied in a rat model of Achilles tendinopathy, and the histological analysis results indicated that CEFFE-MNs had better anti-apoptosis and tendon repair-promoting effects *in vivo* than CEFFE injection therapy. Moreover, the Achilles tendon of rats with tendinopathy regained partial tensile modulus after treatment with CEFFE-MNs. The transcriptome sequencing results showed that the molecular mechanism underlying the anti-inflammatory and anti-apoptotic effects of CEFFE is related to the inhibition of the TNF/NF-κB signaling pathways. In addition, CEFFE-MNs rescued LPS-induced mitochondrial damage *in vitro*. CEFFE also inhibited Drp 1, a protein downstream of TNF signaling, thereby rescuing mitochondrial dynamics and improving mitochondrial function. Our study suggests that the inhibition of TNF/NF-κB signaling pathways by CEFFE-MNs may be a potential mechanism for treating chronic tendinopathy.

Achilles tendinopathy includes chronic and acute Achilles tendon injuries. Studies have shown that the Achilles tendon is in an inflammatory state before the onset of acute rupture, and changes in the collagen composition and structure of tendons in chronic inflammation result in decreased mechanical properties, which increases the incidence of acute Achilles tendon rupture [16,17]. There are several therapies for Achilles tendon disease, among which stem cell therapy is the best treatment, with adipose-derived stem cells ameliorating collagenase-induced tendon lesions in a rat model. Tendon-derived stem cells or autologous bone marrow stem cells also enhance the healing process of Achilles tendon disease in humans and animals; however, their widespread application has been limited [18]. Compared with stem cell therapy, CEFFE has more advantages and application possibilities, with no tumorigenicity or heterotopic ossification, and low requirement for long-term living environments [7,19]. In addition, traditional anti-inflammatory and pro-proliferative drugs can be delivered through MNs [20]; therefore, we delivered CEFFE via MNs in a non-invasive, sustained-release manner to treat Achilles tendinopathy.

Achilles tendinopathy has been treated via the painless and controllable transdermal exosome (EXO) delivery by MNs loaded with tendon stem cell-derived EXO. However, tendon stem cell-derived EXO did not induce an immune response, self-differentiation, or carcinogenicity while inhibiting inflammation and promoting tendon repair to some extent [21]. Moreover, the identification of tendon stem cells and isolation and purification of EXO remain challenging. In contrast, CEFFE is easier to obtain and has good therapeutic effects. Additionally, soluble HA was previously used as the layer material for MNs [15], and a biocompatible GelMA was used as the tip material; however, the basal layer fell off shortly after application to the skin, leaving the GelMA tip in the wound [[17], [22], [23]]. CEFFE is more readily available and has shown antioxidant and anti-inflammatory effects in multiple studies [[9], [10], [11]]. Therefore, in the present study, we encapsulated CEFFE with GelMA to achieve painless, sustained-release drug delivery for the treatment of Achilles tendinopathy.

For the past decade, the role of inflammation in tendinopathy and repair has been controversial, with growing evidence supporting its role in promoting disease progression [24,25]. Targeted inhibition of inflammation after tendon repair improves preclinical outcomes; however, its underlying mechanism remains unclear [19]. The canonical NF-κB pathway constitutes a family of “fast-acting” protein complexes that bind to a dimer-specific inhibitor of NF-κB protein (IκB) and are latent in the cytoplasm [10]. Inflammatory stimuli act through receptor-specific mechanisms, inducing the recruitment of the IκB kinase complex, which phosphorylates IκB, leading to its degradation [16]. These results suggest that CEFFE-MNs might inhibit the excessive activation of the TNF/NF-κB pathway. TNF contains two receptors TNFR 1 and TNFR 2; TNFR 1 is expressed in all tissues and is the primary TNF receptor whereas TNFR 2 is mainly expressed in the immune and hematopoietic cells. TNFR1 mediates the activation of the downstream NF-κB signaling pathway and regulates inflammatory response and apoptosis [9]. In the present study, transcriptomic sequencing showed that CEFFE significantly inhibited LPS-induced cell inflammation and TNF signaling activation. As TNF is a leading inflammatory factor, we further investigated its mRNA and protein levels to verify that CEFFE regulates the inflammatory response and apoptosis downstream of the TNF/NF-κB signaling pathway. A recent clinical study has suggested that NF-κB signaling is involved in the early stages of Achilles tendinopathy [26]. A recent study showed that the IκB kinase (IKKβ), which mediates NF-κB activation, promotes tendinopathy progression in mice. Treatment with an NF-κB inhibitor blocks NF-κB signaling and proinflammatory cytokine production [20]. The IKK complex, which is indispensable for NF-κB signaling, consists of a scaffold protein and catalytic subunits IKKα and IKKβ, which regulate inflammation by targeting IκBα [20,25]. IL-1β and TNF are both frequently studied inflammatory factors, and they are not exactly the same. TNF is usually activated by LPS and expressed in monocytes or macrophages. Effector cells (tendon cells, etc.) are stimulated by TNF to activate inflammatory pathways. On the one hand, TNF activates NF-kB signal to activate gene transcription; on the other hand, TNF induces inflammatome formation and IL-1β expression [19,26]. Second, we focused on the central role of NF-kB in inflammation and in tendinopathy [18], which is activated by TNF rather than IL-1β. However, my personal opinion is that we are looking at different locations of the inflammatory pathway in tendinopathy. Of course, we have discussed the similarities and differences between supplementing TNF and IL-1β in tendinopathy inflammation for the reviewer's reading [[27], [28]]. In the present study, we found that the mitochondria of tendon cells in an inflammatory state exhibited functional and morphological changes. Mitochondrial membrane permeability is often critical for apoptosis and increased mitochondrial outer membrane permeability releases mitochondrial proteins that activate caspases, leading to apoptosis [[29], [30], [31], [32]]. In this study, we found that CEFFE alleviates mitochondrial damage both *in vitro* and *in vivo* via the NF-κB pathway. However, further molecular mechanistic studies are required to verify the relationship between mitochondrial damage and NF-κB pathway activity during the treatment of tendon inflammation with CEFFE.

In the present study, we observed that the chronically inflamed Achilles tendon adhered to the surrounding tissue *in vivo*. Tissue adhesions manifest as excessive accumulation of extracellular matrix components, such as Col3 and α-smooth muscle actin, which surround damaged tissue, leading to tissue fibrosis, pain, and dysfunction [31]. Persistent inflammation activates tissue fibrosis and is a major contributor to many fibrotic diseases. NF-κB is a key regulator of inflammation and cell survival. Among the five subunits of the NF-κB complex, P65 is a key member of the canonical NF-κB pathway. The fibrotic role of P65 in many diseases has been previously studied; core pathways are conserved in many fibrotic diseases, suggesting that P65 may be an integral factor in tendon adhesions [33,34]. The healing of injured tendons follows the classic wound-healing process, including inflammation, cell proliferation, and apoptosis [34]. Therefore, P65, a key signaling molecule in the NF-κB pathway, may regulate tendon repair and fibrosis. The correlation between P65 and tendon adhesion has been analyzed in human samples and confirmed in a rat model of tendinopathy [31]. In the present study, we found that the surface of the Achilles tendon in a rat model of chronic tendinopathy was rough, and it was difficult to separate it from the Achilles tendon and surrounding tissues due to adhesions in the *in vivo* study. Moreover, although injection-only therapy alleviated collagenase I-induced chronic tendon inflammation, the sustained-release administration of MNs was more effective for tendinopathy. We also found that LPS-induced tendon cell inflammation promotes the activation of the NF-κB pathway. Moreover, treatment with CEFFE downregulated the expression of p-P65, a key regulator of the NF-κB pathway *in vivo*. However, we lacked a more detailed and quantitative evaluation of Achilles tendon adhesions in each group of rats in our study. As tendon adhesion is an important component of tendinopathy treatment that directly affects tendon function, CEFFE should be further explored in follow-up studies, including tendon adhesions and the overall exercise capacity of individuals, to identify potential tendon adhesion and tendinopathy prevention methods.

## 5 Conclusions

The present study evaluated a sustained-release MNs drug delivery system for repairing chronic tendinopathy by loading CEFFE. The *in vitro* and *in vivo* experiments showed that CEFFE-MNs had anti-apoptotic and tendon repair-promoting effects with enhanced biosafety. Mechanistically, CEFFE-MNs played an anti-apoptotic and therapeutic role in mitochondrial damage by inhibiting the TNF/NF-κB signaling pathways. The CEFFE-MNs we designed not only rescued the histological phenotype of Achilles tendinopathy in rats but also significantly improved the mechanical strength of Achilles tendon. Therefore, these results suggest that CEFFE-MNs have great potential for clinical translation.

## Credit author statement

**Tianyou Kan:** Conceptualization, Investigation, Formal analysis, Writing-original draft. **Zhaoyang Ran:** Investigation, Formal analysis, Writing original draft. **Lin Sun:** Investigation, Formal analysis. **Xu Jiang**: Investigation, Formal analysis. **Lingli Hou:** Investigation, Formal analysis. **Yiqi Yang:** Methodology. **Zhuoxuan Jia:** Investigation. **Wenjie Zhang:** Methodology, Methodology, Supervision, Funding acquisition. **Liao Wang:** Conceptualization, Methodology, Supervision. **Mengning Yan:** Conceptualization, Methodology, Supervision, Funding acquisition, Writing-review & editing. **Kai Xie:** Conceptualization, Supervision, Funding acquisition, Writing-review & editing.

## Funding

This work was supported by the 10.13039/501100001809National Natural Science Foundation of China (82202680 and 12272232), the 10.13039/501100003399Science and Technology Commission of Shanghai Municipality (22YF1422900 and 21S31905500), 10.13039/100007219Natural Science Foundation of Shanghai (20ZR1432000), the Shanghai Collaborative Innovation Program on Regenerative Medicine and Stem Cell Research (2019CXJQ01), Cross-disciplinary Research Fund of Shanghai Ninth People's Hospital, 10.13039/501100004921Shanghai Jiao Tong University School of Medicine (JYJC202201) and Transverse Research Project (202117013).

## Declaration of competing interest

The content is solely the responsibility of the authors. All authors have read and approved the manuscript and have no conflicts of interest to disclose. The funding body was not involved in the design, collection, analysis, and interpretation of data, or the writing of the manuscript.

## Data Availability

Data will be made available on request.
