# Supplementary material for: Cell-free fat extract-loaded microneedles attenuate inflammation-induced apoptosis and mitochondrial damage in tendinopathy
Source: Mater Today Bio. 2023 Aug 1;22:100738. doi: 10.1016/j.mtbio.2023.100738 (PMC10433131; doi:10.1016/j.mtbio.2023.100738)
Supplement: Multimedia component 1 [file mmc1.docx]

**Supplementary Figures**


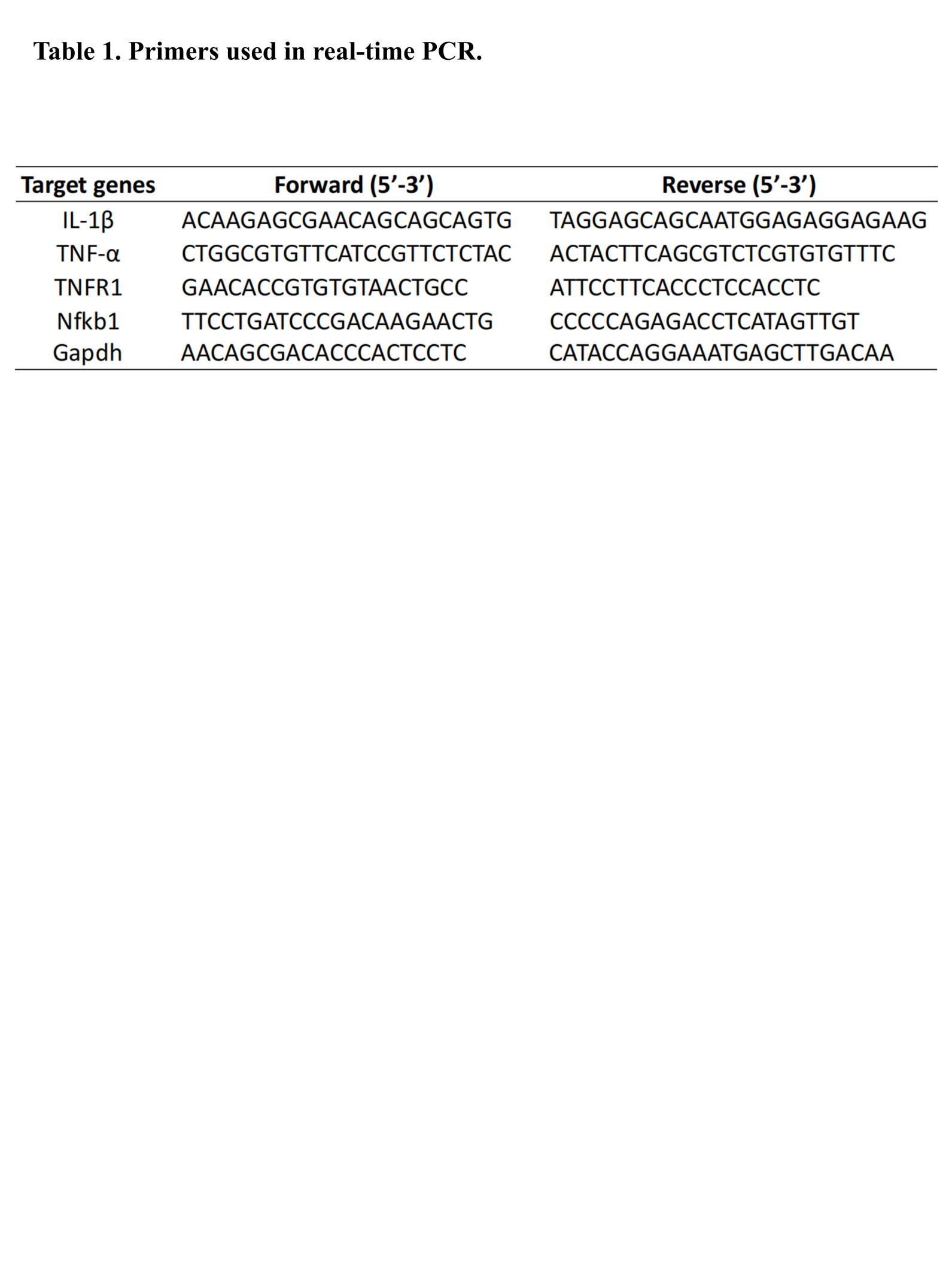


**Table 1.** Primers used in real-time PCR.


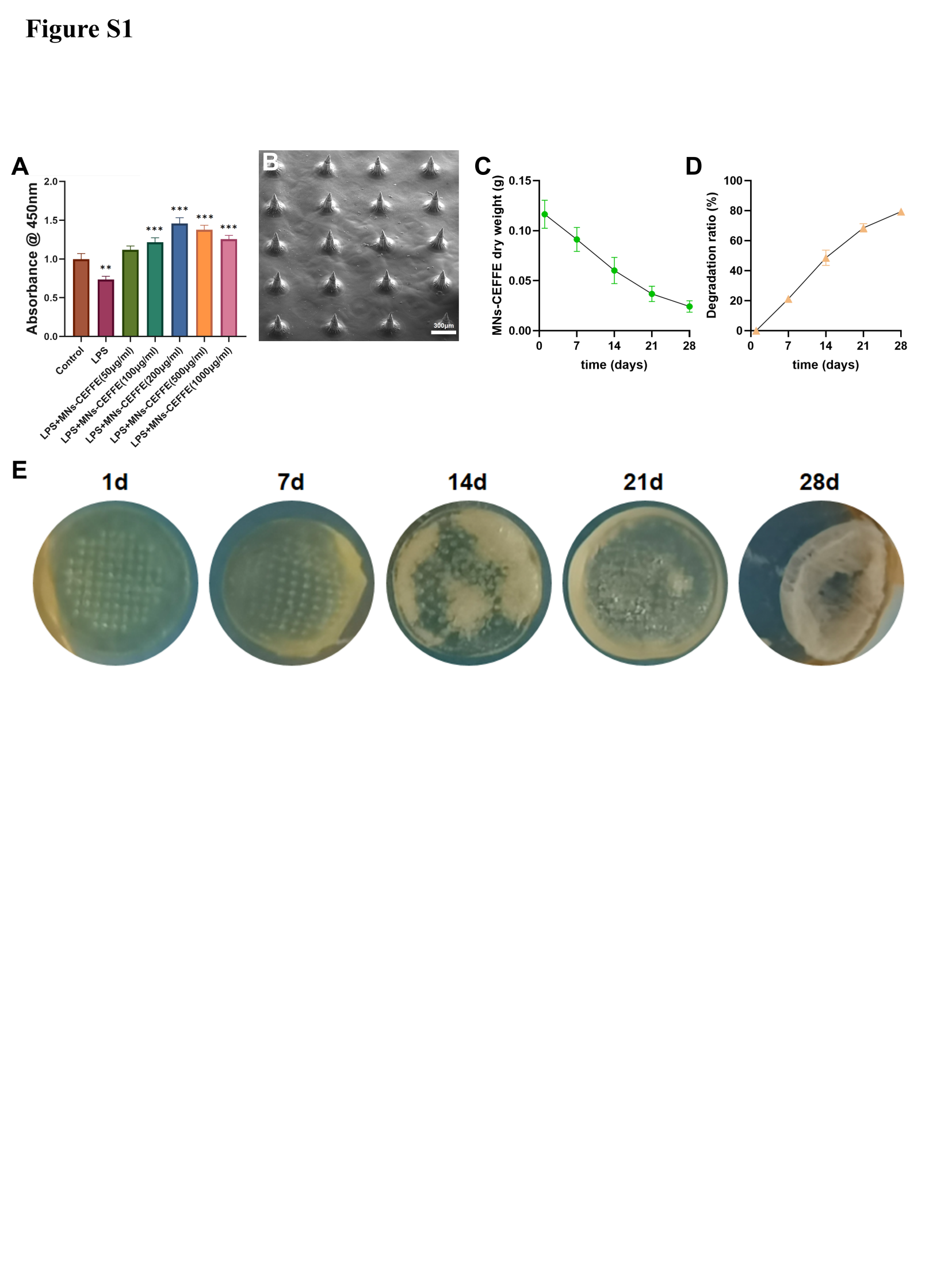


**Figure S1.** (A) Cytotoxicity of LPS-induced tendon cells by different concentrations of CEFFE. (B) SEM view of CEFFE-MNs. (C,D) Degradation testing of CEFFE-MNs. (E) General views of CEFFE-MNs after degradation at 1, 7, 14, 21 and 28 days.


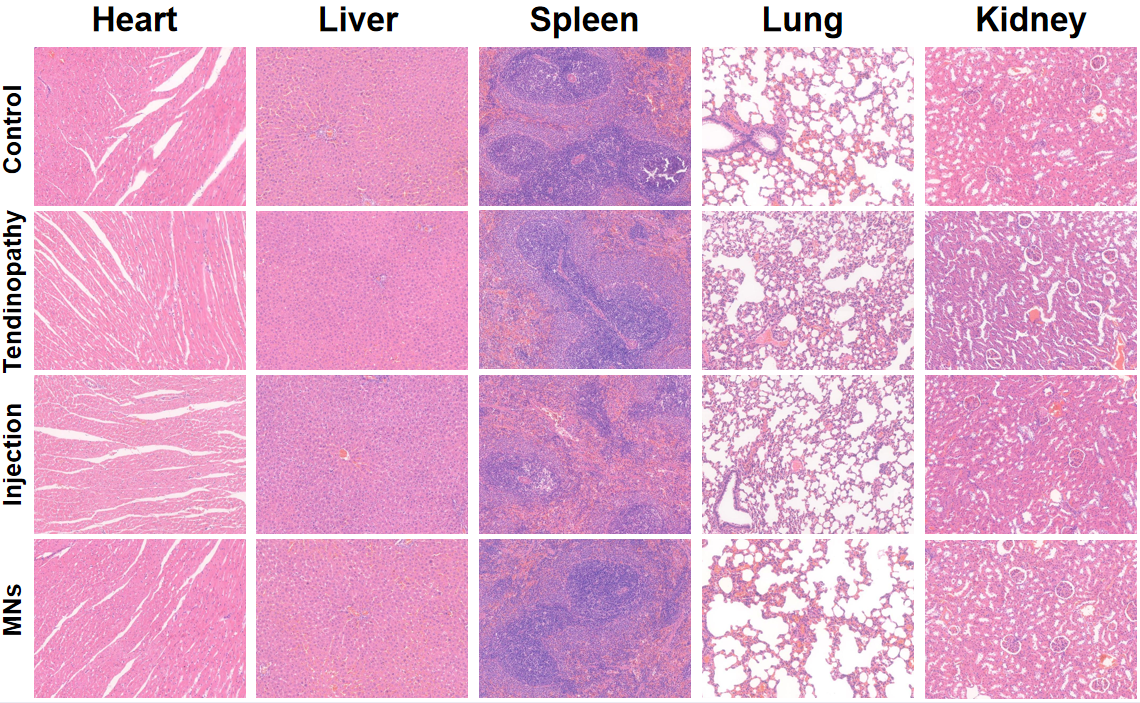


**Figure S2.** Evaluation of organizational microstructures and toxicity of the heart, liver, spleen, lung, and kidney in the four groups with HE staining (100 × magnification).


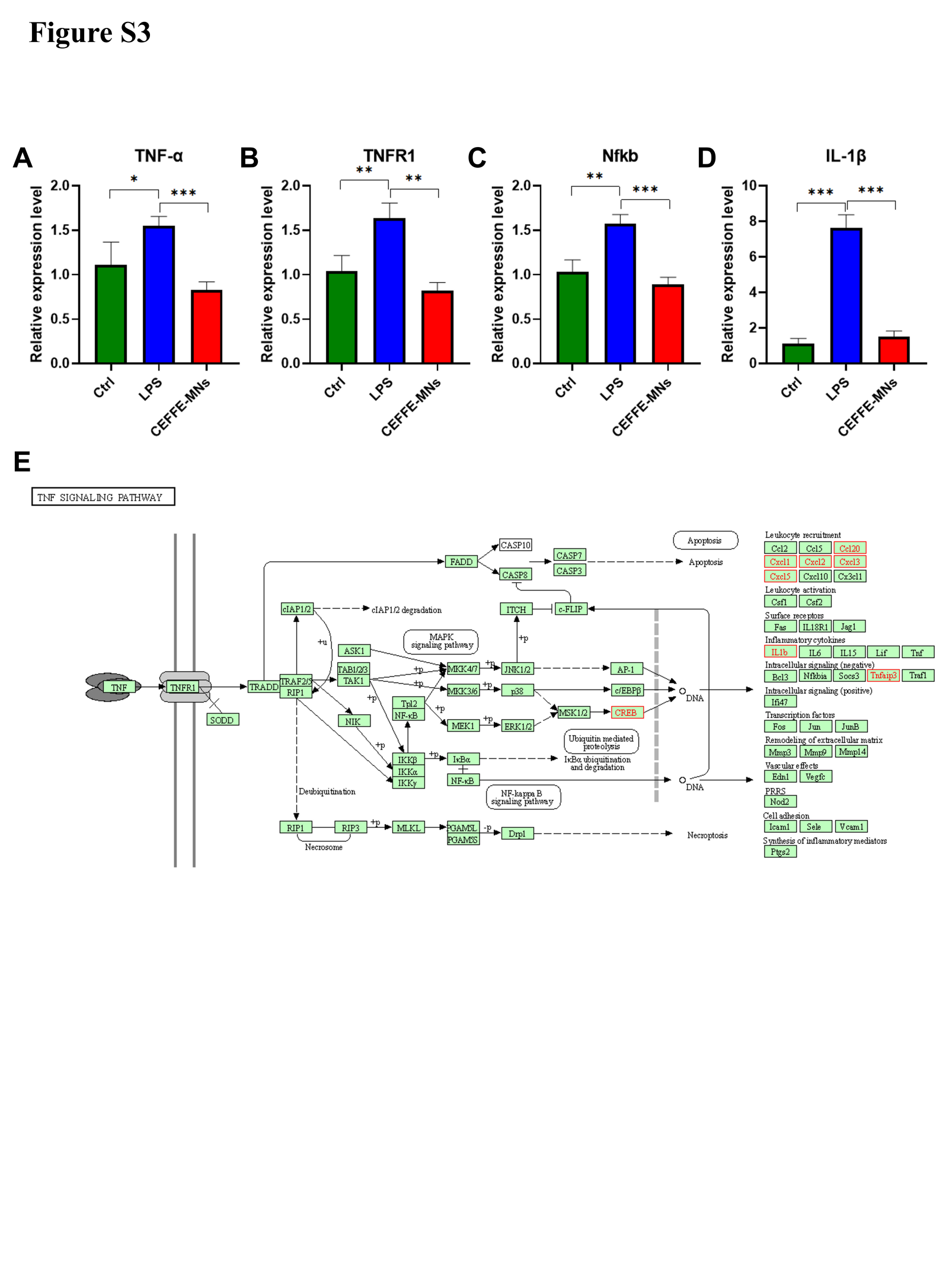


**Figure S3.** (A-D) RT-PCR of TNF-α, TNFR1, Nfkb and IL-1β in the control group, LPS group and CEFFE group (N=3). Data are shown as means ± SD, *P < 0.05, **P < 0.01, ***P < 0.001.
